# Effects of Microbial Aerosol in Poultry House on Meat Ducks' Immune Function

**DOI:** 10.3389/fmicb.2016.01245

**Published:** 2016-08-17

**Authors:** Guanliu Yu, Yao Wang, Shouguo Wang, Changmin Duan, Liangmeng Wei, Jing Gao, Tongjie Chai, Yumei Cai

**Affiliations:** ^1^College of Animal Science and Veterinary Medicine, Shandong Agricultural University, Sino-German Cooperative Research Centre for Zoonosis of Animal Origin Shandong ProvinceTai'an, China; ^2^Deizhou People's HospitalDeizhou, China; ^3^The Central Hospital of Tai'anTai'an, China

**Keywords:** microbial aerosol, immune indicators, stress, cherry valley ducks, poultry houses

## Abstract

The aim of this study was to evaluate effects of microbial aerosols on immune function of ducks and shed light on the establishment of microbial aerosol concentration standards for poultry. A total of 1800 1-d-old cherry valley ducks were randomly divided into five groups (A, B, C, D, and E) with 360 ducks in each. To obtain objective data, each group had three replications. Concentrations of airborne bacteria, fungi, endotoxin in different groups were created by controlling ventilation and bedding cleaning frequency. Group A was the control group and hygienic conditions deteriorated progressively from group B to E. A 6-stage Andersen impactor was used to detect the aerosol concentration of aerobes, gram-negative bacteria, fungi, and AGI-30 microbial air sampler detect the endotoxin, and Composite Gas Detector detect the noxious gas. In order to assess the immune function of meat ducks, immune indicators including H5 AIV antibody titer, IgG, IL-2, T-lymphocyte transformation rate, lysozyme and immune organ indexes were evaluated. Correlation coefficients were also calculated to evaluate the relationships among airborne bacteria, fungi, endotoxin, and immune indicators. The results showed that the concentration of airborne aerobe, gram-negative bacteria, fungi, endotoxin have a strong correlation to H5 AIV antibody titer, IgG, IL-2, T-lymphocyte transformation rate, lysozyme, and immune organ indexes, respectively. In addition, when the concentration of microbial aerosol reach the level of group D, serum IgG (6–8 weeks), lysozyme (4 week) were significantly higher than in group A (P < 0.05); serum IL-2 (7 and 8 weeks), T-lymphocyte transformation rate, lysozyme (7 and 8 weeks), spleen index (6 and 8 weeks), and bursa index (8 week) were significantly lower than in group A (*P* < 0.05 or *P* < 0.01). The results indicated that a high level of microbial aerosol adversely affected the immune level of meat ducks. The microbial aerosol values in group D provide a basis for recommending upper limit concentrations of microbial aerosols for healthy meat ducks.

## Introduction

The air in poultry houses is usually heavily contaminated by large quantities of airborne microorganisms, endotoxins and toxic gases (NH_3_, H_2_S), etc. (Nimmermark et al., 2009; Cambra-López et al., 2010; Lawniczek-Walczyk et al., 2013). In airborne microorganisms, there is a high concentration of non-pathogenic microorganisms leading to animal immunosuppression (Douwes et al., 2003; Fiegel et al., 2006). The high level of airborne aerobe could reduce animal immunity and growth rate (Wolinsky, 2006). Many studies have documented that exposure to fungal aerosol may be associated with asthma, acute toxic and allergic, and it may threaten caretakers and external ambient in animal houses as well (Bush and Portnoy, 2001; Pavan and Manjunath, 2014). The percentage of airborne gram-negative bacteria in the bacterial aerosol is small, but it contains a lot of pathogenic bacteria (Zucker et al., 2000). Endotoxins are ubiquitous in the environment. They are a biologically active lipopolysaccharide that is a component of the outer membrane of gram-negative bacteria (Balasubramanian et al., 2012). According to Pirie, inhaled endotoxin contributes significantly to the induction of airway inflammation and dysfunction (Pirie et al., 2003). Many occupational studies have shown positive associations between endotoxin exposure and respiratory disorders including asthma-like syndrome, chronic airway obstruction, organic duct toxic syndrome, byssinosis, bronchitis, etc. (Madsen, 2006). Zucker et al. have used it as an important symbol of organic dust in the air of poultry house (Zucker et al., 2000). Endotoxin also affects human humoral and cellular immunity (Burrell, 1990). Furthermore, in terms of toxic gases in animal house, ammonia and hydrogen sulfide are two well-known toxic components (Yao and Li, 2010). They can cause respiratory, eye diseases and even poisoning death (Teye et al., 2008; Yao and Li, 2010; Barrasa et al., 2012).

To date, numerous correlation studies have focused on microbial aerosol composition, concentration and mechanisms of spread to the surrounding ambient (Zucker et al., 2000; Madsen, 2006; Duan et al., 2007; Masclaux et al., 2013; Matković et al., 2013). However, studies of microbial aerosol on immune function have not been found. Therefore, the aim of this study was to clarity the effect of microbial aerosol on the immune function of ducks, which was based on comparing the significance between control group and the treatment groups of ducks' specific immune indexes (e.g., IgG, H5 AIV antibody titer, IL-2, etc.) and the non-specific immune factors (e.g., lysozyme), as well as the relationship between major microbial aerosol concentration and immune indicators. Moreover, this study also could enlighten future studies on the establishment of microbial aerosol concentration standards for poultry breeding.

## Materials and methods

### Experimental design

This study was conducted at the Animal Husbandry & Veterinary Station of Shandong Agricultural University, China during January–March, 2014. Five groups were set up, with a control group A and 4 treatment groups (B, C, D, and E, with hygienic conditions deteriorating progressively from group B to E). Each group had three replications with each in a separate poly-tunnel. The poly-tunnel is covered by a double layer of clear plastic with 2 cm insulation in between and with steel or wood arch frames and bedding on the ground. It is naturally ventilated and the duck feces are cleaned manually. All 15 poly-tunnels were identical, equipped with similar exhaust fan, radiator and incandescent light bulb (80 W). Air warmed by the heat from the sun in the day and the bulb at night was retained in the building by the roof and walls. Temperature of each group was maintained between 20 and 24°C using radiators and exhaust fan. A regime of 16 h light (between 05:00 and 21:00) and 8 h darkness was used, with a 25 min twilight phase at the end of each day, and light intensity was about 60 lx at bird-eye height. The size of poly-tunnel was 4.0 × 4.0 × 3.0 m, with a window (2.0 × 1.5 m) facing the sun. A glass door (0.8 × 1.8 m) was used to observe the behavior of ducks in each poly-tunnel. A total of 1800 1-day old cherry valley ducks were placed in the ducks houses, with 360 ducks in each poly-tunnel. The ducks were reared on the floor with thick bedding (wood-shavings), and food and water were automatically refilled. Phosphoric acid (H_3_PO_4_), calcium superphosphate [Ca(H_2_PO_4_)_2_], ferrous sulfate (FeSO_4_·7H_2_O), caustic lime (CaO), acticarbon and alum [Al_2_(SO_4_)_3_·18H_2_O] were used to absorb noxious gases (such as NH_3_; Moore et al., 1996; Do et al., 2005; Yao and Li, 2010). Before the trial began, environmental management measures under rearing conditions in poly-tunnels in North China were investigated, while health management measures in different groups were examined. Based on these findings, the health management measures of all treatment groups in this experiment are listed (Table 1; Yu et al., 2016). All animal experiments were performed according to the guidelines of the Committee on the Ethics of Animals of Shandong and the appropriate biosecurity guidelines, and the protocol was approved by Shandong Agricultural University Animal Care and Use Committee (No. SDAUA-2014-066).

**Table 1:** **Management regimes in different groups**.

**Groups**	**Ventilation method**	**Ventilation time (h)**	**Frequency of troughs cleaning, sterilization, and bedding replacement**
A (Control)	Natural and mechanical	24	Once/day
B (Treatment)	Mechanical	24	Once/2 days
C (Treatment)	Mechanical	18	Once/3 days
D (Treatment)	Mechanical	12	Once/4 days
E (Treatment)	Mechanical	10	Once/5 days

### Sample collection and analysis

#### Determination of airborne aerobe, fungi, and gram-negative bacteria

A 6-stage Andersen impactor (airflow 28.3 L/min), at a height of about 0.2 m (duck's breathing zone) above the ground in the central part of each poly-tunnel, was used to sample airborne aerobe, fungi and gram-negative bacteria weekly at 7:00, 14:00, and 20:00 h, respectively. The samples were selected three times for 1–2 min a time in every poly-tunnel. Soy agar medium with 5% defibrinated sheep blood, Sabouraud's medium (HB0253-8, Hope Bio-Technology Co., Ltd, Qingdao, China) and a gram-negative bacteria selective medium (HB8643, Hope Bio-Technology Co., Ltd, Qingdao, China) were used as sampling media for aerobes, fungi, and gram-negatives, respectively. For Sabouraud's medium, after high temperature steam sterilization, add Chloramphenicol (dose is 0.2 g/L) into it. The samples were taken to the microbe laboratory and cultured in incubators (aerobic condition)–the aerobes at 37°C for 1 day, fungi at 25°C for 4 days and gram-negative bacteria at 37°C for 3 days. After incubation, the numbers of colonies on plates were determined with a Colony Star counter and concentrations were expressed as colony forming units per m^3^ (CFU/m^3^; Andersen, 1958).

#### Determination of airborne endotoxin

Air samples for endotoxin were collected by the AGI-30 Sampler (airflow 12.5 L/min) weekly at the height of 0.2 m (Duck's breathing zone) for 20 min, with 50 mL pyrogen-free water as media (Brachmann et al., 1964). Sampling sites were set in the central part of each poly-tunnel. Endotoxin content was determined by Limulus amebocyte lysate (LAL) assay (QLC2100 Bio Whittaker, Walkersville, MD, USA). A standard curve obtained from an *Escherichia* was used to express concentrations as endotoxin units (EU) which were presented as EU/m^3^.

#### Determination of noxious gas

Noxious gas was detected by Composite Gas Detector (GC310, Chicheng Electric Co., Ltd, Henan, China) in all groups in real time. The instrument was hung 0.2 m above the ground on the wall. The concentration of noxious gas was presented as mg/Kg.

#### Determination of immune indicators

At the age of 10 days, ducks were immunized with H5 AIV vaccine (Reassortant Avian Influenza Virus H5 Subtype Vaccine, Inactivated Strain Re-6+Strain Re-4, Qingdao Yebio Biological Engineering Co., Ltd., Qingdao, China) by neck subcutaneous, 1.5 mL of each one.

Five mL of blood sample was collected in EDTA vacuum tubes through vena digitalis from each duck of 4-, 5-, 6-, 7- and 8-week old (60 ducks in each group). After centrifugation for 10 min at 800 g, serum samples were stored at −20°C until analysis. Duck IgG detection kit, duck IL-2 detection kit (both of them were purchased from Nanjing SenBenJia Biological Technology Co., Ltd. Nanjing, China), lysozyme detection kit (Nanjing Jiancheng Bioengineering Institute, Nanjing, China) and hemagglutination-inhibition (HI) test were used to detect the serum IgG, IL-2, lysozyme and H5 AIV antibody titer, respectively.

MTT (Methy Thiazolyl Tetrazolium) colorimetric assay was used to detect T lymphocytes transformation rate (Lazar et al., 2010; Hsiao and Huang, 2011; Yin et al., 2015). The procedure as follows:

One mL of blood sample was collected in EDTA vacuum tubes through vena digitalis from each duck of 4-, 5-, 6-, 7-, and 8-week old (60 ducks in each group).

One mL whole blood dilution (Shanghai Yanjin Biotechnology Co.Ltd. Shanghai, China) was added to the above blood sample (1 mL), then mixed. The mixture was added on 4 mL lymphocyte separation fluid (Beijing Dingguo Chengsheng Biotechnology Co., Ltd. Beijing, China) for 15 min centrfugation at 800 g. The white coat that under the plasma layer was sucked out and washed 2 times with 3–5 times volume RPMI 1640 culture liquid (Sigma, USA) without calf serum, each time with centrfugation for 10 min at 800 g.

Counting with Trypan Blue (Sigma, USA), the living cells was more than 95%. Single cell suspensions (final concentration was 3 × 10^6^ /mL) were prepared by RPMI 1640 complete culture liquid containing 10% calf serum. The single cell suspension was cultured in a cell incubator at 37°C, 6.5% CO_2_ for 14 h. Peripheral blood lymphocyte were obtain and then prepared for lymphocyte suspension (final concentration was 2 × 10^6^ /mL).

Cells are grown in microtiter plates (tissue culture grade, 96 wells, flat bottom). 100 μL of the lymphocyte suspension and 100 μL of the PHA (phytohaemagglutinin; Beijing Baiaosentai Biotechnology Co., Ltd. Beijing, China; final concentration was 25 μg / mL) was added (final concentration was 2 × 10^6^ /mL) into each test well. 100 μL of the lymphocyte suspension and 100 μL of the RPMI 1640 was added into each control well. The replications is five. After the incubation in a cell incubator at 37°C, 6.5% CO_2_ for 44 h, 20 μL of MTT (5 mg/mL) was added into each well and then continued to incubate for 4 h. The supernatant of each well was discarded carefully.

After that, 150 μL of dimethyl sulfoxide was added into each well and then oscillated for 10 min on microoscillator. The value of OD 570 nm was measured by Microplate Reader (Antai AY-858, Shanghai, China).

$$\begin{array}{l} \text{T-lymphocyte transformation rate}=\\ \frac{\text{Mean value of test }{\text{OD}}_{570}}{\text{Mean value of control }{\text{OD}}_{570}}\times 100\% \end{array}$$

#### Determination of immune organ indexes

The ducks of 4-, 6-, 8-week were weighed and recorded (60 ducks in each group). After that, thymus, spleen and bursa were collected from those ducks, respectively, and then weighed and recorded. At last, the immune organ indexes were calculated according to the follow formula.

$$\text{Immune organ indexes = }\frac{\text{Immune organ }(\text{g})}{\text{Body weight }(\text{Kg})}\times 100\%$$

### Statistical analysis

Data collection ran from week 4 to week 8. Data for each group were expressed as the mean of three replications. The maximum, minimum and median value were used for the air ambient parameter (Duan et al., 2007). All statistical analyses were performed using SAS 9.1 Software (SAS Institute, Inc., Cary, NC, USA). One-way ANOVA analysis with multiple-range test was used to evaluate the difference among groups (Duncan, 1955). Results are expressed as mean ± standard deviation (SD). Correlation between major microbial concentrations and immune indicators were analyzed by Pearson's. *P* < 0.05 was considered statistically significant.

## Results

### The concentrations of microbial aerosol and noxious gas under different hygienic conditions

Over time, the concentrations of airborne aerobe, airborne fungi, airborne gram-negative, airborne endotoxin, and NH_3_ showed an overall trend of increase with the deteriorating of hygienic conditions, however, concentration of NH_3_ in each group was lower than the poultry harmless criterion (10 ppm, GB/T 18407.3–2001), and H_2_S was not found in all groups (Table 2; Yu et al., 2016).

**Table 2:** **Airborne aerobe, airborne fungi, airborne gram-negative bacteria, airborne endotoxin, and noxious gas concentrations under different hygienic conditions**.

**Parameter**	**Value**	**Groups**				
		**A**	**B**	**C**	**D**	**E**
Aerobe (×10E5 CFU/mE3)	Minimum	0.46	0.69	0.68	0.59	0.71
	Maximum	2.30	5.10	5.76	5.96	8.96
	Mean	1.05	2.45	2.94	**2.96**	4.31
Fungi (×10E4 CFU/mE3)	Minimum	0.11	0.21	0.19	0.85	0.78
	Maximum	3.49	3.54	3.95	5.73	8.05
	Mean	1.02	1.32	1.44	**2.63**	3.07
Gram-negative bacteria (×10E4 CFU/mE3)	Minimum	0.20	0.32	0.36	0.89	0.98
	Maximum	2.04	1.95	3.62	8.87	5.03
	Mean	0.93	1.24	1.68	**3.09**	2.64
Endotoxin (×10E3 EU/mE3)	Minimum	0.20	0.40	0.28	0.13	0.56
	Maximum	25.6	72.4	102.4	144.8	144.8
	Mean	6.49	10.48	23.03	**41.78**	47.79
NH_3_ (mg/Kg)	Minimum	0	0	2	4	4
	Maximum	4	12	10	15	14
	Mean	2.56	2.42	5.67	**9.48**	8.97
H_2_S (mg/Kg)	Minimum	–^a^	–	–	–	–
	Maximum	–	–	–	–	–
	Mean	–	–	–	–	–

*All value for total experimental period*.

a *Below the limit of detection*.

*The bold values could be used as a basis for recommending upper limit concentrations of microbial aerosols for healthy meat ducks*.

### The effect of microbial aerosol on specific immunity of meat ducks

#### H5 AIV antibody titer

**Figure 1:**
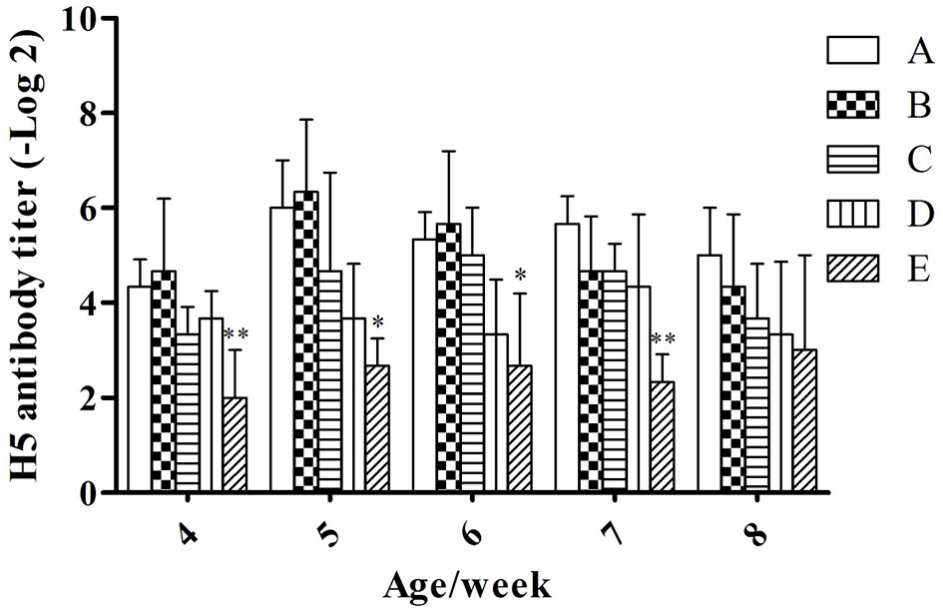
**H5 AIV antibody titer under different hygienic conditions (***n*** = 60)**. Note: The comparison was between treatment groups (B–E) and control group (A) at the same age/week, ^*^P < 0.05 and ^**^P < 0.01. The same as below. ^*^Means that the difference between treatment groups (B–E) and control group (A) was significant.

Under the condition without booster immunization, the H5 AIV antibody titer in serum of meat ducks of groups A and B reached a peak at week 5 (6.00 ± 1.00 and 6.33 ± 1.53, respectively), however, groups C, D, and E reached the peak at weeks 6, 7, and 8 (5.00 ± 1.00, 4.33 ± 1.53, and 3.00 ± 2.00, respectively; Figure 1). At the same week age, with the microbial aerosol concentrations increasing, the concentration of H5 AIV antibody titer in the serum of meat ducks generally showed a tendency of decline.

Serum H5 AIV antibody titers were lower in groups E than in group A (*P* < 0.01) at weeks 4 and 7; groups E were lower than group A (*P* < 0.05) at weeks 5 and 6.

#### IgG

**Figure 2:**
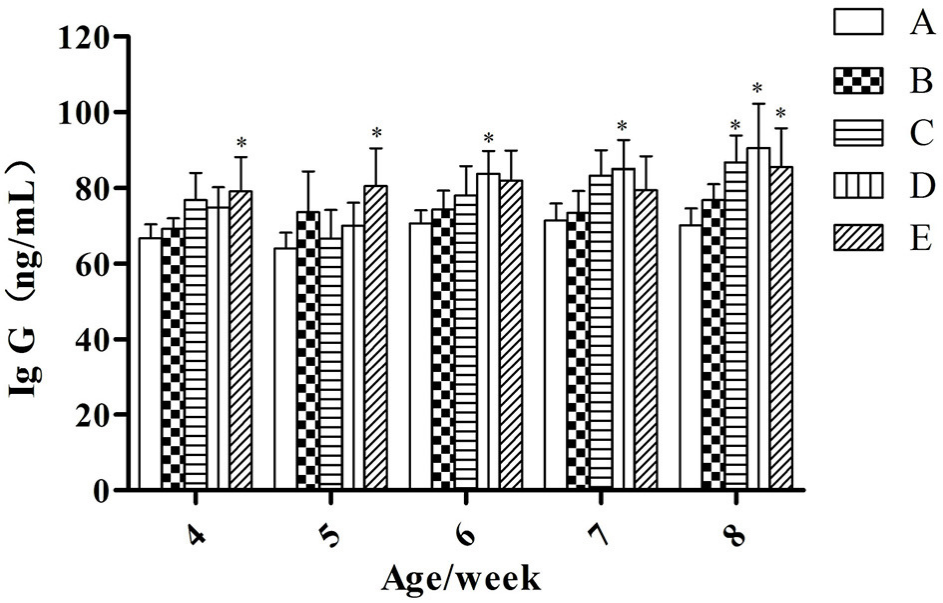
**IgG concentration under different hygienic conditions (***n*** = 60)**. ^*^Means that the difference between treatment groups (B–E) and control group (A) was significant.

At the same week age, with the increase of the microbial aerosol concentrations, the concentration of IgG in serum of meat ducks generally showed a tendency of increase (Figure 2).

Concentration of serum IgG were higher in groups E than in groups A (*P* < 0.05) at week 4 and 5; groups D were higher than group A (*P* < 0.05) at week 6 and 7; groups C, D, and E were higher than group A (*P* < 0.05) at week 8.

#### IL-2

**Figure 3:**
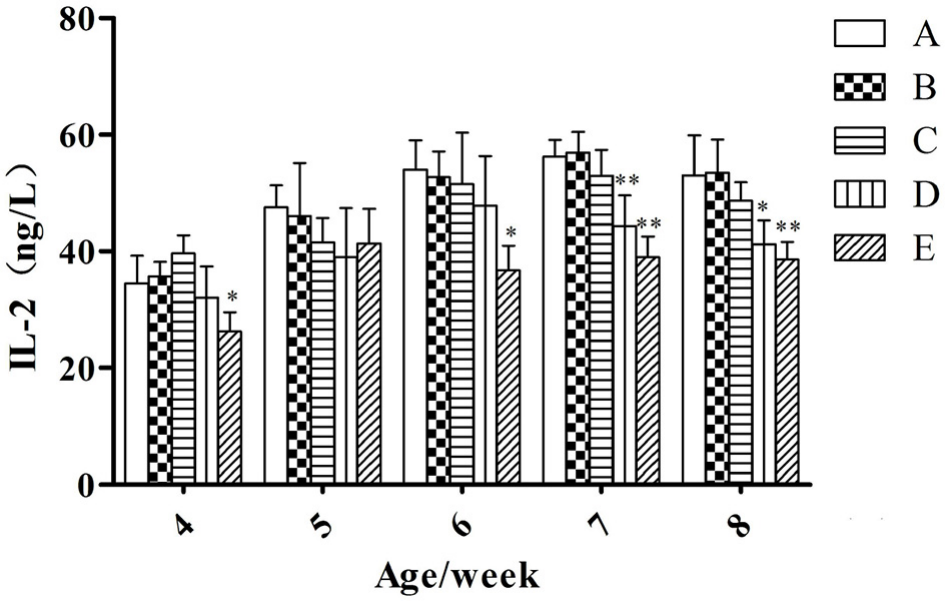
**IL-2 concentration under different hygienic conditions (***n*** = 60)**. ^*^Means that the difference between treatment groups (B–E) and control group (A) was significant. ^**^Means that the difference between treatment groups (B–E) and control group (A) was extremely significant.

At the same week age, with the increase of the microbial aerosol concentrations, the concentration of IL-2 in serum of meat ducks generally showed a tendency of decline (Figure 3).

Serum IL-2 in groups E were lower than in group A (*P* < 0.05) at week 4 and 6; groups D and E were lower than group A (*P* < 0.01) at week 7; groups D and E were lower than group A (*P* < 0.05 and *P* < 0.01, respectively) at week 8.

#### T-lymphocyte transformation rate

**Figure 4:**
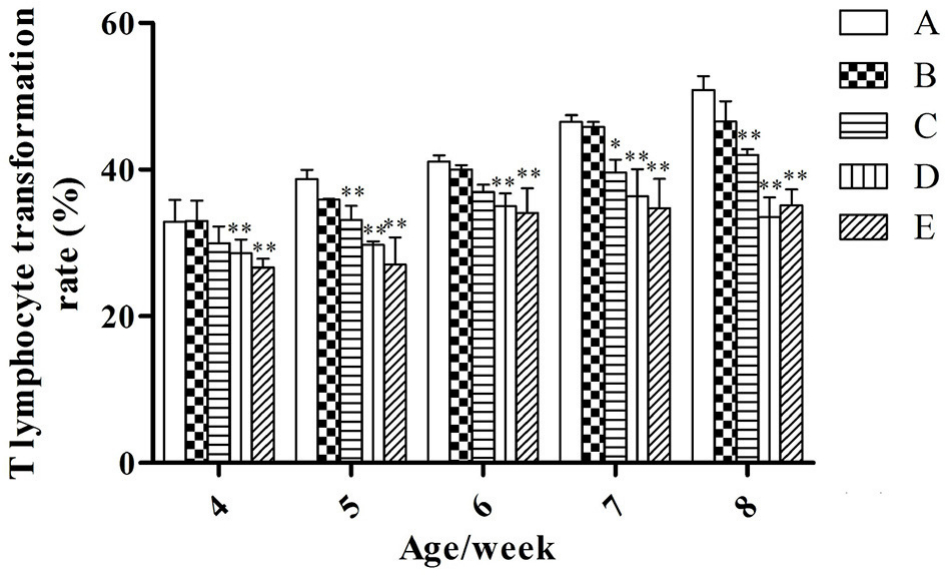
**T-lymphocyte transformation rate under different hygienic conditions (***n*** = 60)**. ^*^Means that the difference between treatment groups (B–E) and control group (A) was significant. ^**^Means that the difference between treatment groups (B–E) and control group (A) was extremely significant.

At the same week age, with the increase of the microbial aerosol concentrations, T-lymphocyte transformation rate of meat ducks generally showed a tendency of decline (Figure 4), and the decline range was obvious.

T-lymphocyte transformation rates of groups D and E were lower than that of group A (*P* < 0.01) at weeks 4 and 6; groups C, D, and E were lower than that of group A (*P* < 0.01) at weeks 5 and 8; groups C, D, and E were lower than that of group A (*P* < 0.05, *P* < 0.01 and *P* < 0.01, respectively) at week 7.

### The effect of microbial aerosol on non-specific immunity of meat ducks

#### Lysozyme

At the same week age, as microbial aerosol concentrations increasing, the concentration of lysozyme in serum of meat ducks generally showed a tendency of increase at first (at weeks 4 and 5) and then showed a tendency of decline (at weeks 6, 7, and 8; Table 3).

**Table 3:** **Lysozyme concentration (U/mL) under different hygienic conditions (***n*** = 60)**.

**Weeks**	**Groups**				
	**A**	**B**	**C**	**D**	**E**
4	120.14 ± 4.22	126.10 ± 4.99	124.60 ± 9.73	130.62 ± 7.99^*^	133.62 ± 6.14^*^
5	148.32 ± 5.21	150.85 ± 5.79	149.22 ± 6.68	152.30 ± 7.76	156.83 ± 9.35
6	153.72 ± 6.77	152.80 ± 11.29	158.16 ± 8.43	145.65 ± 7.03	148.98 ± 9.43
7	176.42 ± 6.43	158.47 ± 6.92^**^	162.38 ± 9.68^**^	151.90 ± 7.56^**^	141.37 ± 5.98^**^
8	166.31 ± 4.46	180.53 ± 6.65^**^	156.90 ± 8.21	144.82 ± 8.60^**^	139.43 ± 7.34^**^

The comparison was between treatment groups (B–E) and control group (A) at the same age/week, ^*^ P < 0.05 and ^**^ P < 0.01. The same as below.

* *Means that the difference between treatment groups (B–E) and control group (A) was significant*.

** *Means that the difference between treatment groups (B–E) and control group (A) was extremely significant*.

Serum lysozyme in groups D and E were higher than group A (*P* < 0.05) at week 4; groups B, C, D and E were lower than group A (*P* < 0.01) at week 7; groups D and E were lower than group A (*P* < 0.01), but the group B was higher than group A (*P* < 0.01) at week 8.

#### Immune organ indexes

At the same week age, with the increase of the microbial aerosol concentrations, the index of thymus, spleen and bursa of meat ducks generally showed a tendency of decline (Table 4).

**Table 4:** **Immune organ indexes under different hygienic conditions (***n*** = 60)**.

**Items**	**Weeks**	**Groups**				
		**A**	**B**	**C**	**D**	**E**
Thymus index	4	2.81 ± 0.17	2.60 ± 0.11	2.49 ± 0.63	2.16 ± 0.33	2.09 ± 0.51
	6	2.44 ± 0.47	2.16 ± 0.38	1.95 ± 0.13	1.98 ± 0.48	1.80 ± 0.34
	8	2.21 ± 0.45	2.19 ± 0.28	1.96 ± 0.26	1.56 ± 0.33	1.57 ± 0.41
Spleen index	4	1.32 ± 0.12	1.24 ± 0.26	1.25 ± 0.33	1.03 ± 0.38	0.96 ± 0.08
	6	1.26 ± 0.22	1.06 ± 0.14	0.90 ± 0.14	0.79 ± 0.28^*^	0.69 ± 0.20^*^
	8	1.19 ± 0.21	0.96 ± 0.08	0.98 ± 0.18	0.80 ± 0.20^*^	0.72 ± 0.1^**^
Bursa index	4	1.84 ± 0.39	1.74 ± 0.56	1.86 ± 0.17	1.49 ± 0.47	1.54 ± 0.33
	6	1.44 ± 0.08	1.27 ± 0.16	1.31 ± 0.25	1.16 ± 0.20	1.19 ± 0.19
	8	1.46 ± 0.23	1.16 ± 0.16	1.03 ± 0.20^*^	0.97 ± 0.23^*^	0.89 ± 0.23^*^

* *Means that the difference between treatment groups (B–E) and control group (A) was significant*.

** *Means that the difference between treatment groups (B–E) and control group (A) was extremely significant*.

For thymus index, there was no significant effect (*P* > 0.05). As for spleen index, groups D and E were lower than group A (*P* < 0.05) at week 6; groups D and E were lower than group A (*P* < 0.05 and *P* < 0.01, respectively) at week 8. For bursa index, groups C, D and E were lower than group A (*P* < 0.05) at week 8.

#### Relationships between microbial aerosol constituents and immune indicators

The correlation between microbial aerosol and immune indicators is shown in Table 5.

**Table 5:** **Correlation between concentrations of major microbial aerosol components and values of immune indicators**.

**Immune index**	**Aerobe**	**Fungi**	**Gram-negative bacteria**	**Endotoxin**
IgG	*r* = 0.91^*^	*r* = 0.86	*r* = −0.90^*^	*r* = 0.86
H5 AIV antibody titer	*r* = −0.90^*^	*r* = −0.95^*^	*r* = −0.87	*r* = −0.98^**^
IL-2	*r* = −0.84	*r* = −0.99^**^	*r* = −0.88^*^	*r* = −0.99^**^
T-lymphocyte trans-formation rate	*r* = −0.89^*^	*r* = −0.95^*^	*r* = −0.95^*^	*r* = −0.95^*^
Lysozyme	*r* = −0.79	*r* = −0.97^**^	*r* = −0.95^*^	*r* = −0.97^**^
Thymus index	*r* = −0.92^*^	*r* = −0.96^**^	*r* = −0.83	*r* = −0.98^**^
Spleen index	*r* = −0.94^*^	*r* = −0.96^**^	*r* = −0.92^*^	*r* = −0.95^*^
Bursa index	*r* = −0.88^*^	*r* = −0.93^*^	*r* = −0.94^*^	*r* = −0.89^*^

Significant relationships (

* *P < 0.05*,

** *P < 0.01) expressed as Pearson correlation coefficients (**r**) in bold*.

The concentration of aerobe showed a strong correlation to IgG, H5 AIV antibody titer, T-lymphocyte transformation rate, Thymus Index, Spleen Index and Bursa Index (*r* = 0.91 at *P* < 0.05, *r* = −0.90 at *P* < 0.05, *r* = −0.89 at *P* < 0.05, *r* = −0.92 at *P* < 0.05, *r* = −0.94 at *P* < 0.05, *r* = −0.88 at *P* < 0.05, respectively).

As for fungi, a significant negative correlation was recorded between fungi and H5 AIV antibody titer, IL-2, T-lymphocyte transformation rate, Lysozyme, Thymus Index, Spleen Index and Bursa Index (*r* = 0.95 at *P* < 0.05, *r* = −0.99 at *P* < 0.01, *r* = −0.95 at *P* < 0.05, *r* = −0.97 at *P* < 0.01, *r* = −0.96 at *P* < 0.01, *r* = −0.96 at *P* < 0.01, *r* = −0.93 at *P* < 0.05, respectively).

The concentration of endotoxin revealed the same dependency on H5 AIV antibody titer, IL-2, T-lymphocyte transformation rate, Lysozyme, Thymus Index, Spleen Index, and Bursa Index (*r* = 0.98 at *P* < 0.01, *r* = −0.99 at *P* < 0.01, *r* = −0.95 at *P* < 0.05, *r* = −0.97 at *P* < 0.01, *r* = −0.98 at *P* < 0.01, *r* = −0.95 at *P* < 0.05, *r* = −0.89 at *P* < 0.05, respectively).

However, the gram-negative bacteria correlated negatively with IgG, IL-2, T-lymphocyte Transformation Rate, Lysozyme, Spleen Index, and Bursa Index (*r* = −0.90 at *P* < 0.05, *r* = −0.88 at *P* < 0.05, *r* = −0.95 at *P* < 0.05, *r* = −0.95 at *P* < 0.05, *r* = −0.92 at *P* < 0.05, *r* = −0.94 at *P* < 0.05, respectively).

The prediction models are as follows:

Y = 75.49 − 2.78 × 10E-6 X_1_ + 2.13 × 10E-5 X_2_, *R*^2^ = 0.3414, *p* = 0.5086 > 0.05
Y: IgG (ng/mL); X_1_: airborne aerobe (CFU/mE3); X_2_: airborne gram-negative bacteria (CFU/mE3)
Y = 4.76 + 2.41 × 10E-6 X_1_ + 1.00 × 10E-4 X_2_ − 1.03 × 10E-4 X_3_, *R*^2^ = 0.9760, *p* = 0.0230
Y: H5 AIV antibody titer (−Log 2); X_1_: airborne aerobe (CFU/mE3); X_2_: airborne fungi (CFU/mE3); X_3_: airborne endotoxin (EU/mE3)
Y = 55.61 − 5.01 × 10E-4 X_1_ + 6.01 × 10E-6 X_2_ − 1.03 × 10E-4 X_3_, *R*^2^ = 0.8795, *p* = 0.0141
Y: IL-2 (ng/mL); X_1_: airborne fungi (CFU/mE3); X_2_: airborne gram-negative bacteria (CFU/mE3); X_3_: airborne endotoxin (EU/mE3)
Y = 43.77 − 5.53 × 10E-6 X_1_ + 1.01 × 10E-4 X_2_ + 3.69 × 10E-6 X_3_ − 3.03 × 10E-4 X_4_, *R*^2^ = 0.8417, *p* = 0.0392
Y: T-lymphocyte transformation rate (%); X_1_: airborne aerobe (CFU/mE3); X_2_: airborne fungi (CFU/mE3); X_3_: airborne gram-negative bacteria (CFU/mE3); X_4_: airborne endotoxin (EU/mE3)
Y = 155.45 − 8.35 × 10E-6 X_1_ − 1.99 × 10E-6 X_2_ − 2.00 × 10E-4 X_3_, *R*^2^ = 0.9097, *p* = 0.0517 > 0.05
Y: Lysozyme (U/mL); X_1_: airborne fungi (CFU/mE3); X_2_: airborne gram-negative bacteria (CFU/mE3); X_3_: airborne endotoxin (EU/mE3)
From the analysis above, it could be concluded that the concentration of airborne aerobe, fungi, gram-negative bacteria, endotoxin have a strong correction with the value of H5 AIV antibody titer, IgG, IL-2, T-lymphocyte rate, lysozyme, and immune organ indexes, respectively. Thus, it can provide a substantial evidence to confirm the effect of microbial aerosol on immune level.

## Discussion

Microbial aerosol originates from feed, manure, litter, as well as microorganisms, their byproducts and fragments in poultry houses (Millner, 2009; Just et al., 2011). Airborne aerobes, fungi, gram-negative bacteria and their bioproducts or biological fragments (such as endotoxins) are major components (Yu et al., 2016). The concentrations and components of it could reflect the condition of ambient sanitation in animal houses (Zucker and Muller, 2000; Kaliste et al., 2002). High concentrations of microbial aerosol and its metabolites (endotoxin, NH_3_, H_2_S, etc) are important factors affecting the health and productivity of animals (Prazmo et al., 2003; Banhai et al., 2008).

In this study, four treatment groups with gradually deteriorating hygienic conditions and one control group under standard hygienic sanitary management were set up by changing the frequency of trough cleaning, sterilization, bedding replacement, and ventilation (Table 1). The concentrations of airborne aerobes, fungi, gram-negative bacteria, and endotoxin in groups B, C, D increased both over time and as hygienic conditions deteriorated (Table 2). The results show that routine hygienic management measures, such as ventilation, bedding replacement and sterilization can reduce bioaerosols in duck poly-tunnels, which is important in order to maintain optimal microclimate and hygiene. Phosphoric acid, calcium superphosphate, ferrous sulfate, caustic lime, acticarbon, and alum were effective in absorbing noxious gases.

To the best of our knowledge, this study is the first to evaluate the effects of microbial aerosol on duck immunity. As we all known, immunity of animal can be divided into specific and non-specific immunity, and specific immunity can be divided into humoral and cellular immunity. In order to explore the effects of microbial aerosol on the immune function of duck. We chose IgG, H5 AIV antibody titer as reliable indicators for humoral immunity; took IL-2, T-lymphocyte transformation rate as representativeness indexes for cellular immunity; and took lysozyme, Immune organ indexes as non-specific immune factors.

Immunity to avian influenza is mainly based on humoral immunity, and detection of antibody titer of avian influenza contributes to indicating the condition of specific immune system protection (Ellis et al., 2004; Liu et al., 2006). As for serum IgG, it is the highest level of immunoglobulin in the blood of bodies. The activity of anti-bacteria, anti-virus and anti-toxin of IgG can be embodied in animal blood, and it plays essential roles in “Main Immune” (Borghesi et al., 2014). IL-2, also called T-cell growth factor, is the main cytokine in regulating cellular immune (Bayer et al., 2013). It is mainly produced by activated T-lymphocytes, and also can activate a variety of immune cells, regulate the body's immunity and enhance the body's anti-inflammatory effects, etc. (Song et al., 2005). In the process of the immune response, T-lymphocyte transformation rate is involved in the cellular immune response, therefore, it is often used to assess the functional status of lymphocytes and the status of body's immunity (Toivanen and Toivanen, 1973; Hovi et al., 1978; Kim et al., 1996).

Lysozyme is a kind of hydrolase that has special effects on the microbial cytoderm, which relaxes cytoderm and loses the protective effect on cells, and results in bacteria dissolution eventually (Sung et al., 2011). In the process of anti-bacterial infection, lysozyme often used as an important indicator that reflects strength of non-specific immunity (Fiolka et al., 2012; Zhao et al., 2014). The weight of thymus, spleen and bursa can be used to evaluate the immune status of poultry. It also reflects the strength of immune function intuitively (Rivas and Farbricant, 1985).

Over the experimental period, as microbial aerosol concentration increasing, serum IgG and lysozyme (4 and 5 weeks) increased, whereas H5 AIV antibody titer, IL-2, T-lymphocyte transformation rate, lysozyme (6 and 8 weeks), and immune organ indexes decreased. When the concentration of microbial aerosol reach the level of group D, serum IgG (6–8 weeks), serum lysozyme (4 week) were significantly higher than in group A (*P* < 0.05); serum IL-2 (7 and 8 weeks), T-lymphocyte transformation rate, serum lysozyme (7 and 8 weeks), spleen index (6 and 8 weeks), and bursa index (8 week) were significantly lower than in group A (*P* < 0.05 or *P* < 0.01).

Microbial aerosols at certain concentrations can stimulate the stress response, and stress can have serious adverse effects on welfare (Yu et al., 2016). Under stress, animals have to activate energy to combat the stressor, which can enhance catabolism and weaken the anabolism of protein and fat. Where animals are reared in environments contaminated with microorganisms, the nutrient status of organs may be compromised (Benson et al., 1993). This might be the reason for the decreasing tendency of immune organ indexes.

Moreover, if the stress in a long period of time, and it could lead to chronic stress, which could lead to cellular immune inhibition (Schedlowski, 1993; Bartolomucci et al., 2003), cutting down the production of IL-2 in serum (McEwen et al., 1997). This might be the reason for the tendency of serum IL-2 and T-lymphocyte transformation rates.

As for the tendency of serum IgG and lysozyme, after the initial increase it later decreased (6–8 weeks; Figure 2 and Table 3), this may be due to the appearance of “malignant stress” in the late stages of this study, that is, under the short-term and mild stress, animals could adapt to it by compensatory reaction, but the long-term stress at any intensity will result in harmful effects, such as deterioration of physiological function in animals, etc. (He et al., 2011).

In addition, high level of microbial aerosol also affected the humoral immunity level of meat ducks, and it not only reduced the H5 AIV antibody titer of ducks, but also delayed the emergence of the antibody titer peak (Figure 1). This result is analogous to that of Witter (1998). Witter argued that weakened immune function reduces the immune protective effect of the vaccine.

## Conclusions

In conclusion, a high level of airborne aerobe, gram-negative bacteria, fungi, and endotoxin adversely affected the immune level of meat ducks. This study indicates that good ventilation, bedding replacement and sterilization can decrease microbial aerosol concentration effectively. The present findings suggest that the microbial aerosol concentrations of group D provide a basis for recommending upper limit concentrations of microbial aerosols for healthy meat ducks.

## Author contributions

GY and YC designed the experiment and completed most of the works. YW, SW, CD, and JG analyzed some test results and collected materials. TC and LW gave experiment instruction. Thank all the authors' contribution to the experiment.

### Conflict of interest statement

The authors declare that the research was conducted in the absence of any commercial or financial relationships that could be construed as a potential conflict of interest. The handling Editor declared a shared affiliation, though no other collaboration, with authors GY, YW, LW, TC, YC and states that the process nevertheless met the standards of a fair and objective review.
